# Dual inhibition of renin-angiotensin-aldosterone system and endothelin-1 in treatment of chronic kidney disease

**DOI:** 10.1152/ajpregu.00425.2015

**Published:** 2016-03-23

**Authors:** Radko Komers, Horacio Plotkin

**Affiliations:** Retrophin, Cambridge, Massachusetts

**Keywords:** angiotensin II, chronic kidney disease, diabetic nephropathy, endothelin, FSGS

## Abstract

Inhibition of the renin-angiotensin-aldosterone system (RAAS) plays a pivotal role in treatment of chronic kidney diseases (CKD). However, reversal of the course of CKD or at least long-term stabilization of renal function are often difficult to achieve, and many patients still progress to end-stage renal disease. New treatments are needed to enhance protective actions of RAAS inhibitors (RAASis), such as angiotensin-converting enzyme (ACE) inhibitors (ACEIs) or angiotensin receptor blockers (ARBs), and improve prognosis in CKD patients. Inhibition of endothelin (ET) system in combination with established RAASis may represent such an approach. There are complex interactions between both systems and similarities in their renal physiological and pathophysiological actions that provide theoretical rationale for combined inhibition. This view is supported by some experimental studies in models of both diabetic and nondiabetic CKD showing that a combination of RAASis with ET receptor antagonists (ERAs) ameliorate proteinuria, renal structural changes, and molecular markers of glomerulosclerosis, renal fibrosis, or inflammation more effectively than RAASis or ERAs alone. Practically all clinical studies exploring the effects of RAASis and ERAs combination in nephroprotection have thus far applied add-on designs, in which an ERA is added to baseline treatment with ACEIs or ARBs. These studies, conducted mostly in patients with diabetic nephropathy, have shown that ERAs effectively reduce residual proteinuria in patients with baseline RAASis treatment. Long-term studies are currently being conducted to determine whether promising antiproteinuric effects of the dual blockade will be translated in long-term nephroprotection with acceptable safety profile.

inhibition of the renin-angiotensin-aldosterone system (RAAS) plays a pivotal role in treatment of chronic kidney diseases (CKD). Inhibitors of the RAAS (RAASis) can slow the progressive decrease in glomerular filtration rate (GFR), reduce proteinuria, and cardiovascular mortality and morbidity in both diabetic and nondiabetic proteinuric kidney diseases. However, despite documented beneficial effects of RAASis, reversal of the course of progressive forms of CKD or at least long-term stabilization of renal function are often difficult to achieve, and many patients still progress to end-stage renal disease (ESRD). New approaches that would broaden the spectrum of available treatments or enhance protective actions of RAASis are needed to improve prognosis in these patients. As indicated by evidence collected over the past two decades, parallel inhibition of the RAAS and endothelin (ET) system may represent such an approach. In this review we will discuss whether there is evidence supporting this view. Basic physiology and pathophysiology of both systems in the kidney have been extensively studied and have been the subject of numerous experimental and clinical reports including excellent reviews. In this paper we will focus only on data relevant for the topic of dual inhibition of both systems in the treatment of kidney disease.

## RAAS-Endothelin-1 Interface in Kidney

## 

### RAAS in renal physiology and pathophysiology.

Main effectors of RAAS, such as angiotensin II (ANG II) or aldosterone, have well-established actions in the kidney and roles in renal pathophysiology (63, 81). In brief, ANG II, acting mostly via AT_1_ receptors, affects practically all renal compartments and cell types. These effects include hemodynamic actions leading to vasoconstriction and elevations of intraglomerular pressure; promoting cell growth and extracellular matrix (ECM) production resulting in glomerulosclerosis and tubulointerstitial fibrosis (TIF); prooxidant and inflammatory actions as well as effects with implications in podocyte pathophysiology and pathogenesis of proteinuria. Similarly, aldosterone has proscelerotic, fibrogenic, and “proteinuric” effects, in addition to its principal roles in the control of sodium/potassium homeostasis and blood pressure (BP) (74, 76). Inhibition of RAAS leads to at least partial suppression of those actions during the development and progression of kidney disease.

### Endothelin-1 in renal physiology and pathophysiology.

Some actions of RAAS effectors, in particular those of ANG II, resemble renal actions of endothelin-1 (ET-1), another peptide implicated in renal pathophysiology, and the most important of ET peptides with respect to renal physiology. ET-1 has been also well established as a player in renal pathophysiology. It is stimulated by numerous factors known to trigger or to contribute to the development of kidney diseases (summarized in Ref. 40). In general, ET-1 acts as a vasoactive peptide, which also stimulates renal cell growth, proliferation, production of ECM, and inflammation (40) and has major impact on tubular function (42). In the following sections we will briefly review actions of ET-1 with respect to individual renal cell types and compartments and point out parallels as well as important differences compared with RAAS effectors.

### Effects of ET-1 in the renal vascular tree.

Similar to ANG II, ET-1 is involved in the control of renal hemodynamics. Actions of ET peptides in the kidney are mediated by ET_A_ and ET_B_ receptors (reviewed in Ref. 42). Both ET_A_ and ET_B_ receptors on vascular smooth muscle cells mediate ET-1-induced vasoconstricton, whereas ET_B,_ localized on endothelial cells, mediates endothelium-dependent vasodilation. The effects of ET-1 on the renal vascular tree are complex and segment specific. Studies in different experimental settings indicate that ET-1 is preferentially a preglomerular vasoconstrictor (summarized in Ref. 42), although this is not a uniform finding, with some species specificity (46). In addition to its effects on vascular tone, the peptide causes endothelial dysfunction, vascular hypertrophy, and remodeling as observed in both hypertensive (3) and normotensive (15) models of kidney disease.

### Glomerular effects of ET-1.

ET-1 and ANG II have similar impact in glomeruli, as it happens with vascular actions. ET-1 has been implicated in mesangial cell contraction, proliferation (72), and ECM production (73). ET-1 actions may result in glomerulosclerosis (discussed in *Endothelin-1 promotes the development of glomerulosclerosis, TIF, and renal inflammation*). There is increasing evidence that ET-1 plays specific roles in podocyte alterations, apoptosis, and loss, nephrin shedding, being implicated in loss of synaptopodin and cytoskeletal rearrangement resulting in foot process effacement, a hallmark of podocytopathies (7, 12, 47, 66) including focal segmental glomerulosclerosis (FSGS) (12, 17). Podocytes are not only targets of ET-1 actions, but also a source of the peptide with deleterious effects on adjacent glomerular endothelial cells, causing mitochondrial dysfunction and production of reactive oxygen species (17).

In the context of podocyte changes and loss of podocytic proteins, Saleh et al. (65, 67) described blood pressure-independent, ET-1-induced enhanced glomerular permeability to albumin both in vitro and in vivo. Altogether, these actions contribute to the proteinuric effects of ET-1. Most of these glomerular actions are mediated via ET_A_ receptors, albeit a recent study suggests the possible pathophysiological role of ET_B_ expressed on podocytes (47). Treatment with ET receptor antagonists (ERAs) (12, 17, 56), or more recently by genetic deletion of ET receptors (ETRKO) in models of podocyte injury, ameliorated cytoskeletal changes and restored podocyte structural integrity in parallel with reduction in proteinuria (47).

### Endothelin-1 promotes the development of glomerulosclerosis, TIF, and renal inflammation.

While in the glomeruli ET-1 actions may result in glomerulosclerosis, in the tubulointerstitial compartment, ET-1 triggers processes leading to TIF, both processes being linked to renal inflammation (78). They are, therefore, discussed together in this section. The role of ET-1 in the above-mentioned processes has been recognized for two decades. As shown by Hocher et al. (32) transgenic mice overproducing ET-1 develop glomerulosclerosis and interstitial fibrosis and inflammation without concurrent hypertension, suggesting that elevation in ET-1 contributes to renal fibrosis.

Most of the evidence about these effects of ET-1 has been provided by numerous studies demonstrating beneficial effects of ET-1 receptor inhibition or ETRKO in models of kidney diseases that lead to glomerulosclerosis and/or TIF. Acting mostly via ET_A_ receptors, ET-1 has been implicated in increased expression and activity of well-established proinflammatory and profibrotic signaling molecules and mediators, including nuclear factor-κB (NFκB) (27, 47, 78), MCP-1, interleukin-6, adhesion molecules (64, 65, 69, 73, 86), transforming growth factor-β (TGF-β) (25, 66, 69, 78), connective tissue growth factor (CTGF) (78), as well as ECM proteins (10, 25, 73, 78). These molecules have also been traditionally implicated in renal pathophysiological actions of ANG II (34, 35, 85) or aldosterone (29, 30, 63). The prosclerotic/fibrogenic and inflammatory actions of ET-1 may occur as part of ANG II signaling (63), or as a direct consequence of stimulation of ET_A_ receptors (65, 73).

### Effects of ET-1 on tubular function.

Importantly, there are also opposing actions of RAAS effectors and ET-1 in the kidney, in particular in their net effect on Na homeostasis and extracellular fluid control.

Endothelin-1 is synthesized by and binds to all tubular segments to regulate ion transport. However, unlike antinatriuretic actions of ANG II or aldosterone, ET-1 acts as a natriureric peptide. The major site of natriuretic and diuretic actions of ET-1 is in the collecting duct (CD), which is also the predominant site of ET-1 tubular production (39). In the CD, ET-1 inhibits epithelial Na channel (ENaC), one of aldosterone-responsive transporters, acting via multiple signaling pathways (28, 38, 59, 70). Adding complexity, aldosterone has been shown to stimulate ET-1 production in the CD (84), raising a possibility that ET-1-induced inhibition of ENaC represents a feedback mechanism to mitigate aldosterone actions. ET-1 also inhibits Na^+^-K^+^-Cl^−^ cotransporter (NKCC2) in thick ascending limb (31) and activates Na^+^/H+ exchanger (NHE3) in the proximal tubule (45, 48), and the latter effect is also involved in the control of acid-base balance (48). The peptide also decreases arginine vasopressin (AVP)-stimulated water transport further enhancing diuretic actions (26, 55).

Most of the evidence suggests that ET_B_ is the main receptor responsible for natriuretic actions of ET-1. However, observations in mice with double ET_A_/ET_B_ knockout in the CD, which display more severe hypertension than ET_B_ knockout mice, indicate a contribution of ET_A_ to ET-1-induced natriuresis (reviewed in Ref. 42). This is further supported by the fact that edema, fluid retention, or even heart failure complicate clinical use of the selective ERAs (50).

### Interactions and cross-talk between RAAS and ET-1.

In addition to similarities in some actions of ET-1 and effectors of RAAS in the pathophysiology of kidney diseases, there are complex interactions and cross-talk between both systems. ANG II stimulates ET-1 release and expression in a variety of cell types including renal cells (23, 24, 43). ET-1 mediates some of the vascular actions of ANG II. ANG II-dependent increases in ET-1 contribute to the vasoconstrictor effects of ANG II in vitro in isolated vascular preparations (16, 79) and in vivo [e.g., in the skin microcirculation in healthy humans (83)]. ET receptor blockade can inhibit the acute vasoconstrictor responses to ANG in vivo, including within the renal circulation (6, 61). In turn, ET-1 stimulates ANG II formation possibly via enhanced action of ACE in vitro in pulmonary endothelial cells (36, 37). Whether ET-1 stimulates ANG II also in nonvascular and renal cells remains to be established.

ET-1 also stimulates aldosterone secretion, as well as zona glomerulosa cell growth and proliferation (52, 53). Nonselective ET receptor blockade decreases plasma aldosterone concentration, zona glomerulosa proliferation, and aldosterone release by zona glomerulosa preparations in rats transgenic for the renin gene (2). Furthermore, in humans with high to normal renin hypertension, ET_A_ or combined ET_A/B_ blockade reduces plasma aldosterone (62).

In contrast to the effects of ET-1 on ANG II and aldosterone, ET-1 appears to inhibit release of renin. ET-1 inhibits renin release from isolated juxtaglomerular apparatus (51, 60). In vivo, low doses of ET-1 that do not produce any significant changes in total peripheral resistance also reduce renin release as observed in anesthetized dogs (49, 57). Higher doses of ET-1 that increase BP increase plasma renin activity (57). However, it is questionable whether this observation is physiologically relevant considering high BP-induced adaptations in renal hemodynamics that may stimulate renin release. The relationship between ET-1 inhibiting the rate-limiting step in the RAAS activation cascade and parallel enhancement of the actions of its downstream effectors remains unknown. Some authors speculate that ET-dependent inhibition of renin release promotes sodium excretion as part of natriuretic physiological functions of ET-1 (42).

Interactions of ET-1 with another effector of RAAS, angiotensin-(1–7), have been also described in experimental settings (9), but their clinical relevance remains to be established and will not be a subject of this review.

## Does Experimental Evidence Support Superior Effects of Dual RAAS-ET-1 Inhibition in Treatment of Kidney Disease Compared With Monotherapies With RAAS or ET-1 Antagonists?

Altogether, there is persuasive evidence for similarities of actions of RAAS effectors and ET-1 in processes that trigger or perpetuate renal injury. This provides rationale for testing possible additive effects of dual inhibition of both systems in treatment of kidney disease.

The concept of dual inhibition of ANG II and ET-1 in nephroprotection is not new and has been developing since the 1990s. Benigni et al. (8) first studied dual blockade in passive Heyman nephritis, a model of idiopathic membranous nephropathy. The combination treatment with angiotensin-converting enzyme inhibitor (ACEI) trandolapril and ET_A_ antagonist LU-135252 had no additive effects on BP compared with trandolapril alone, but the combination was the only treatment that induced significant reduction of proteinuria compared with untreated passive Heyman nephritis animals, whereas the antiproteinuric effect ET_A_ inhibitor or ACEI in monotherapies was not significant. The combination also prevented the rise in serum creatinine (S-Cr) consistent with superior preservation of kidney function at the end of the follow-up (8 mo). Unlike the monotherapies, the combination attenuated the development of glomerulosclerosis. Amann et al. (1) combined ERA LU-135252 with angiotensin receptor blocker (ARB) fonsartan or ACEI ramipril and compared their effects to each monotherapy in 5/6th nephrectomized rats, a model of secondary FSGS. The combination of ERA with ARB was most effective in preventing the development of TIF, albeit this study did not find significant differences between the animals treated with the combination and ACEI or ARB monotherapies on proteinuria, BP, and glomerulosclerosis. Similarly, atrasentan combined with ACEI trandolapril or losartan was more effective than monotherapies in another hypertensive model, 5/6th nephrectomized rats with overexpression of renin gene, when administered immediately after the kidney mass reduction (14).

In experimental diabetic nephropathy, Gagliardini et al. (25) evaluated the effect of simultaneous inhibition of ET-1 with avosentan and ACEI lisinopril in uninephrectomized STZ-diabetic rats. The treatments were applied in monotherapy or in combination between 4 to 8 mo after streptozotocin (STZ) injection. Combined therapy corrected proteinuria, ameliorated several markers of tubulointerstitial damage, and induced regression of glomerular lesions, while only a partial renoprotection was achieved by each drug alone. The combination restored the number of podocytes while monotherapies only limited podocyte depletion. Defective nephrin expression in diabetes was prevented by both drugs. Altered glomerular size selectivity to large macromolecules of diabetic rats was remarkably improved by lisinopril and the combined treatment. Avosentan ameliorated changes in peritubular capillary architecture and reduced tubular damage score, interstitial inflammation as well as expression of TGF-β and collagen III.

It should be noted that not all studies support physiologically relevant additive effects of ERAs and RAASis. In a study by Cao et al. (13), ACEI perindopril or ARB irbesartan, or their combination, markedly attenuated development of hypertension, proteinuria, loss of GFR, renal structural changes, and molecular markers of nephropathy such as TGF-β or collagen IV mRNA expression in 5/6th nephrectomized rats. These beneficial effects were not observed in rats treated with s BMS193884 or with a nonselective ET-1 receptor blocker bosentan, unlike more recent studies by Amman et al. (1). Moreover, ET-1 antagonists did not enhance any of the above-mentioned protective actions of RAASis. In Zucker diabetic fatty rats, a model of Type 2 diabetes (87), ACEI ramipril was more effective compared with ERA sitaxsentan in reduction of BP, albuminuria, glomerulosclerosis score, renal collagen III expression, and markers of intrarenal inflammation. The study failed to demonstrate any additive protective effects with either agent in this model, albeit the authors conclude that adding ERA to ACEI slightly enhances antiinflammatory actions of ACEI. Finally, as very recently observed, the superior protective effects of dual blockade in 5/6th nephrectomized Ren-2 rats discussed above (14) were not apparent when the treatment was administered several weeks after kidney mass reduction (V. Certikova-Chabova, personal communication).

## Effects of Dual Inhibition in Humans With CKD

Practically all clinical studies published thus far in this field have add-on designs, in which ERAs is added to baseline treatment with ACEIs or ARBs. Most of the studies have focused on reduction of residual proteinuria in patients with baseline RAASis treatment.

Additive effects on proteinuria were initially noted in acute studies with ERAs in patients with nondiabetic CKD caused by various forms of glomerulonephritis and secondary FSGS. Infusion of BQ123 reduced proteinuria more than nifedipine (21), despite similar effects on BP. This effect was more prominent in patients pretreated with ACEIs or ARBs compared with ACEIs alone (22). The authors later reported a more prolonged observation (6 wk) in a similar cohort of patients with primary and secondary nondiabetic glomerulopathies and baseline treatment with ACEIs or ARBs (20). The patients received sitaxsentan, nifedipine, or placebo. Unlike placebo, add-on sitaxsentan further decreased proteinuria, BP, and induced changes in renal hemodynamics characterized by lower GFR and no change in renal plasma flow (RPF) resulting in a substantial decrease in filtration fraction (FF). Except comparable reduction in BP, the patients treated with nifedipine did not demonstrate changes in proteinuria or renal hemodynamics. These data suggested hemodynamically mediated antiproteinuric effect of sitaxsentan.

In a study by Weber et al. (80), investigators focused on the effects of ERA darusentan in patients with resistant hypertension, but the treatment-induced changes in albuminuria were also determined. The study is relevant since 96–99% of patients were in parallel treated with ARBs or ACEIs. Type 2 diabetes was present in about 40% of patients. Darusentan was administered at three dose levels (100, 200, and 300 mg/day) for 14 wk. The two higher doses lead to a significant reduction in albuminuria compared with placebo-treated patients who received only RAASis.

The antiproteinuric effects of dual blockade have been thus far best studied in patients with diabetic nephropathy. In initial studies, ERA avosentan was used to assess the effects of ERA on residual proteinuria. In a short-term (12 wk) phase 2a trial, avosentan (5–50 mg/day) reduced albuminuria by up to 40% compared with placebo in Type 2 diabetic patients with nephropathy, who were in most cases (∼95%) treated with maximal doses of RAASis (82). The effect was independent of the avosentan dose. In contrast to substantial antiproteinuric effect, there were no changes in BP in avosentan-treated patients. Dose-dependent fluid retention was noted as major adverse event associated with avosentan. The ASCEND (A Randomised, Double Blind, Placebo Controlled, Parallel Group Study to Assess the Effect of the Endothelin Receptor Antagonist Avosentan on Time to Doubling of Serum Creatinine, End Stage Renal Disease or Death in Patients With Type 2 Diabetes Mellitus and Diabetic Nephropathy) trial (50) was designed to study avosentan in a large cohort of diabetic patients with maximal doses of RAASis with a focus on hard endpoints such as composite of doubling of S-Cr, initiation of renal replacement therapy and death. The trial was stopped prematurely after 4–5 mo because of excess cardiovascular adverse events associated with avosentan treatment (25 and 50 mg/day), mainly congestive heart failure and fluid overload. Yet, the trial confirmed strong beneficial effect of avosentan on residual proteinuria, reaching 40% further reduction compared with placebo.

More recent studies in this area have been conducted with atrasentan, a highly selective ERA. In a phase 2a study, the effects of atrasentan (0.25, 0.75, and 1.75 mg/day) on albuminuria compared with placebo were assessed for 8 wk in 89 Type 2 diabetic subjects with diabetic nephropathy and moderate albuminuria receiving stable doses of RAASis (41). Reduction in albuminuria was observed in patients treated with higher doses of atrasentan (0.75 and 1.75 mg/day) and reached ∼40%, whereas the 0.25 mg/day dose was without any effect. As in previous trials, dose-related occurrence of edema was reported, being 9, 14, 18, and 46% in placebo, 0.25, 0.5, and 1.75 mg/day atrasentan groups, respectively.

The Reducing Residual Albuminuria in Subjects With Diabetes and Nephropathy With AtRasentan (RADAR) trial (18) that followed has been thus far the largest and best documented study assessing dual blockade in proteinuric CKD. The study tested atrasentan at 0.75 and 1.25 mg/day and did not include the highest dose from the previous study (41), in Type 2 diabetic patients with albuminuria [albumin-to-creatinine ratio (ACR) 500-3,500 mg/g] and eGFR within the 30–75 ml/min range treated with maximally tolerated baseline ACEIs or ARBs. In contrast to placebo, both doses of atrasentan were effective in reducing residual albuminuria by about 40%. There was mild increase in body weight, but not in prevalence of edema, and the treatment was in general well tolerated.

In both studies, atrasentan also reduced BP. This modest add-on effect of atrasentan on BP can hardly explain impressive effects on residual proteinuria. On the other hand, rapid return of albuminuria after drug withdrawal (as observed in the atrasentan studies) suggests contribution of the hemodynamic mechanism to the antiproteinuric effect, in accord with a previous report by Dhaun et al. (20), which showed ERA-induced changes in whole kidney renal function parameters suggestive of changes in glomerular hemodynamics. Detailed measurements of renal hemodynamics were not performed in the atrasentan studies and modest changes in FF could have remained undetected. The decrease in albuminuria after initiation of atrasentan treatment was rapid as was the increase to baseline values in this parameter after discontinuation of the treatment. This further supports a renal hemodynamic mechanism of atrasentan antiproteinuric actions in those trials.

For completeness, the list of this type of studies should include a recently published study exploring the effects of dagutril, an inhibitor of neutral endopeptidase and ET-converting enzyme, in albuminuric Type 2 diabetic patients treated with losartan (58). Despite an effect on BP, this treatment did not influence albuminuria over a period of 8 wk. The treatment decreased ET generation, but due to a different mechanism of action it could be hardly compared with more specific ETA inhibition.

## Perspectives and Significance

A spectrum of issues remain be addressed in future or ongoing studies: Most importantly, it remains unknown whether promising signals from the above-discussed studies can be translated into long-term nephroprotection. Moreover, it is not known whether dual blockade is more beneficial than monotherapies on cardiovascular endpoints in patients with CKD.

It has not been well established how much of the antiproteinuric effect is attributable to BP lowering or changes in renal hemodynamics achieved by the combination. Available clinical studies do not offer unequivocal evidence, albeit it could be argued that modest add-on effects of ERAs in most studies on BP can hardly explain impressive effects on residual proteinuria. On the other hand, rapid return of albuminuria after the drug withdrawal as observed in atrasentan studies (18, 41) suggests contribution of a hemodynamic mechanism to the antiproteinuric effect. In addition, direct effects of ET-1 on glomerular permeability discussed in previous sections (65, 67), which are independent of hemodynamic influences, could contribute to both rapid onset of antiproteinuric effect in response to ERAs as well as to rapid return of albuminuria after their withdrawal. This rapid return of albuminuria may also indicate the fact that despite marked reduction in proteinuria, the treatment does not appear to have long-term benefit in preserving renal architecture, emphasizing the need for long-term trials.

Use of RAASis is limited in patients with lower GFR because of increased risk of acute renal failure and hyperkalemia, which may be precipitated by critical decrease in glomerular filtration pressure. Therefore, it will clinically be important to determine whether parallel ET-1 inhibition could attenuate decreases in glomerular pressure induced by RAASis and extend the period during which RAASis could be safely used in patients with low GFR.

Considering the effects of ERAs on aldosterone secretion, it will be also interesting to determine whether the dual blockade could ameliorate aldosterone escape, a phenomenon observed in some patients treated with RAASis and implicated in partial loss of efficiency of ACEIs or ARBs (11).

### SONAR trial.

Some answers, in particular about the potential of dual blockade in long-term nephroprotection, may be provided by the ongoing study of diabetic nephropathy with atrasentan (SONAR) trial (16b). This is a phase 3 prospective, randomized, multicenter, double-blind, parallel, placebo-controlled study of the effects of atrasentan on renal outcomes in Type 2 diabetic subjects with nephropathy. The investigators will randomize more than 4,000 patients with eGFR 25 to 75 ml·min^−1^·1.73 m^−2^ and a ACR ≥ 300 and < 5,000 mg/g, treated with maximal tolerated dose of ARBs or ACEIs, to receive atrasentan 0.75 mg/day or placebo. The double-blind treatment period is estimated to continue for ∼48 mo. The primary endpoint is time to the first occurrence of a component of the composite renal endpoint: doubling of S-Cr or the onset of ESRD (needing chronic dialysis, renal transplantation or renal death). In addition to the primary endpoint, secondary efficacy endpoints will focus on reduction in proteinuria, effects on the rate of decline in eGFR, and cardiovascular endpoints such as nonfatal myocardial infarction or stroke.

### Dual AT_1_ and ET_A_ receptor antagonists.

Studies discussed thus far had uniformly applied add-on design to test dual RAAS and ET-1 inhibition, i.e., a combination of individual receptor blockers. In addition to this approach, investigators have been developing dual AT_1_ and ET_A_ receptor antagonists, i.e., single molecules that can inhibit both receptors. The synthesis of dual ANG II and ET receptor antagonists was based on recognition of the structural similarities between irbesartan (an ARB) and some ET_A_ receptor antagonists (such as BMS 193884) (54)–a resemblance of the core biphenyl framework of these ET_A_ antagonists to the biphenyltetrazole core characteristic for a spectrum of AT_1_ receptor antagonists, including irbesartan. It was hypothesized that merging the structural elements of these two antagonists would yield a compound with dual activity for both receptors. This strategy led to the design, synthesis, and discovery of potent and orally active antagonists of both AT_1_ and ET_A_ receptors (dual action receptor antagonists, DARA). Detailed synthetic process and chemistry of these compounds has been described in the literature (44, 54). These DARA inhibited ^125^I-labeled Sar-Ile-ANG II binding to rat or human AT_1_ receptors and ^125^I-labeled ET-1 binding to rat or human ET_A_ receptors in a concentration-dependent manner. The compounds were inactive against human AT_2_ and ET_B_ receptors as well as many other G protein-coupled receptors, enzymes, and ion channels.

**Fig. 1.**
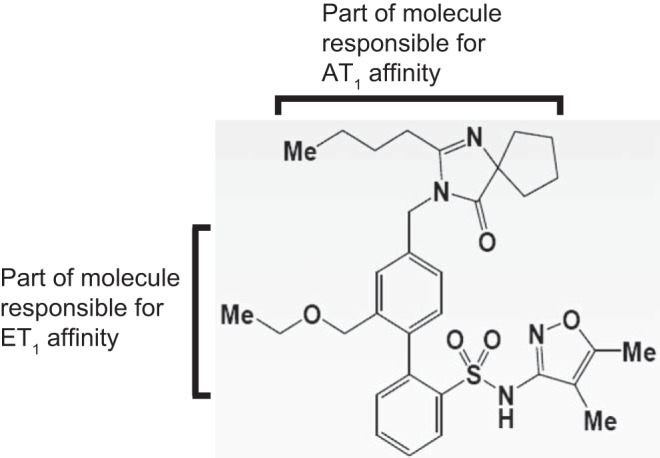
Chemical structure of sparsentan. The figure shows chemical structure of sparsentan with parts of the molecule important for affinities to AT1 and ETA receptors. Adapted with permission (44).

Kowala et al. (44) reported inhibition constants (*K*_i_), calculated from the IC_50_ values, for inhibiting ^125^I-labeled Sar-Ile-ANG II binding to AT_1_/AT_2_ receptors and ^125^I-labeled ET-1 binding to ET_A_/ET_B_ receptors. *K*_i_ of one of these compounds, later called sparsentan (Fig. 1), for human AT_1_ receptor was 0.8 ± 0.1 and for human ET_A_ receptor 9.3 ± 1.1, whereas the affinity toward AT_2_ and ET_B_ receptors was negligible (>1,000). For comparison, the values for *K*_i_ of irbesartan for the *K*_i_ of irbesartan for AT_1_ was 1.1 ± 0.3. Further evidence for the dual antagonistic action of DARA compounds was demonstrated by their ability to reduce acute increases of blood pressure in rats caused by bolus intravenous infusions of either ANG II or BigET-1 (54). Moreover, the DARA were more effective in reducing blood pressure compared with equimolar dose of irbesartan in models of hypertension such as spontaneously hypertensive rats and Dahl salt-sensitive rats (44).

Sparsentan was further developed by Pharmacopeia and Ligand as a promising antihypertensive agent. There were seven phase 1 clinical studies performed in healthy volunteers and two phase 2 studies in patients with essential hypertension. The results of these studies have not been published. In brief, although the focus of those studies were not on proteinuria, they were helpful to understand the generally positive safety profile of the drug and its effects on hypertension, which were stronger than those of irbersartan.

### DUET trial.

Efficacy of sparsentan is currently explored in the DUET trial, a randomized, double-blind, active-control, dose-escalation study evaluating the antiproteinuric efficacy and long-term safety of sparsentan in patients with primary FSGS (16a). The study has been designed to test the hypothesis that in these patients, sparsentan lowers proteinuria to a greater degree than an ARB irbesartan alone while maintaining a favorable drug safety profile. Patients, age 8–75 yr old, with baseline eGFR > 30 ml/min and Up/c > 1 g/g with primary or genetic forms of FSGS are eligible to participate in the study. The patients are randomized after 2 wk washout of RAASis to receive either sparsentan (200, 400, and 800 mg/day) or irbesartan (300 mg/day) as an active control. This double-blind period will last for 8 wk followed by an open-label phase treatment with sparsentan (144 wk) to assess long-term safety. The primary endpoint is change in protein-to-creatinine ratio (Up/c) from baseline to 8 wk postrandomization. Secondary objectives will focus on metabolic effects of sparsentan, RAAS activity, long-term safety, and quality of life.

### Other potential targets for dual RAAS and ET-1 inhibition.

Experimental evidence together with emerging case reports suggest that there is a spectrum of disorders that might be particularly responsive to dual inhibition but have not yet been systematically tested in clinical trials. These conditions include renal involvement in sickle cell anemia (3, 75) and scleroderma crisis (19, 33, 71). IgA nephropathy appears also as a promising new target. A special note should be reserved to the potential of dual blockade in preeclampsia. Both ANG II and ET-1 have been implicated in pathogenesis of this disorder (68, 77). However, because of teratogenic potential, ARBs, ACEIs, and in particular, ET-1 receptor antagonists, are contraindicated during pregnancy. The solution might be provided by the development of inhibitors that do not cross the placental barrier.

**Fig. 2.**
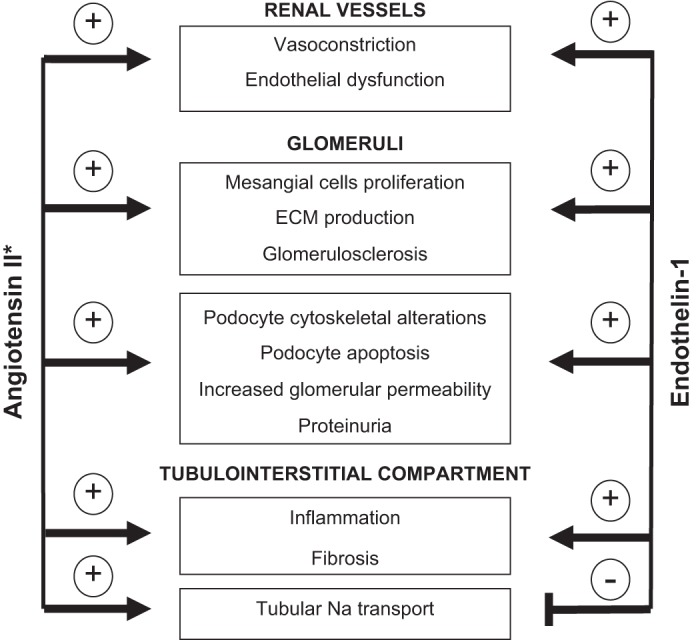
Schematic presentation of major similarities and differences in physiological and pathophysiological actions of angiotensin II and endothelin 1 in the kidney. Both peptides have similar impact on processes implicated in renal pathophysiology. In contrast they differ in their role in the control of tubular transport. More detail in text. *Applies also to aldosterone.

In conclusion, dual inhibition of the RAAS and endothelin ET_A_ receptor emerges as a promising approach to enhance the spectrum of beneficial effects of RAASis in patients with CKD (Fig. 2). In addition to the theoretical rationale for combination suggested by basic research, persuasive clinical evidence currently exists for additive antiproteinuric effect of the dual blockade in patients with diabetic nephropathy. Whether these promising effects could be translated into long-term nephroprotection, i.e., preservation of kidney function and renal architecture, suggested by some experimental studies, needs to be further tested in appropriately designed clinical trials. These trials should also focus on long-term safety, in particular with respect to fluid retention, and on the impact of dual blockade on cardiovascular disease, the major cause of mortality in patients with CKD. The SONAR trial is expected to provide some important data in this context. In the clinical arena, there is still little evidence about the therapeutic potential of dual blockade in nondiabetic proteinuric kidney diseases. This gap is being currently filled by ongoing DUET trial in primary FSGS, but application of the dual blockade in a spectrum of other indications that have been shown to be responsive separately to RAAS or ET-1 inhibition still warrants clinical testing.

## DISCLOSURES

Both authors are employed by Retrophin, Inc. This company is clinical developing sparsentan, a dual angiotensin and endothelin receptor antagonist, for the potential treatment of primary FSGS.

## AUTHOR CONTRIBUTIONS

Author contributions: R.K. prepared figures; R.K. and H.P. drafted manuscript; R.K. and H.P. edited and revised manuscript; R.K. and H.P. approved final version of manuscript.

## References

[1] Amann K, Simonaviciene A, Medwedewa T, Koch A, Orth S, Gross ML, Haas C, Kuhlmann A, Linz W, Scholkens B, Ritz E Blood pressure-independent additive effects of pharmacologic blockade of the renin-angiotensin and endothelin systems on progression in a low-renin model of renal damage. J Am Soc Nephrol 12: 2572–2584, 2001. 11729225 10.1681/ASN.V12122572

[2] Andreis PG, Rebuffat P, Neri G, Rossi GP, Nussdorfer GG Effects of irbesartan and bosentan on the blood pressure and adrenal zona glomerulosa function in heterozygous transgenic TGR[mREN2]27 rats. Life Sci 67: 543–547, 2000. 10993119 10.1016/s0024-3205(00)00648-2

[3] Ataga KI, Derebail VK, Archer DR The glomerulopathy of sickle cell disease. Am J Hematol 89: 907–914, 2014. 24840607 10.1002/ajh.23762 PMC4320776

[6] Balakrishnan SM, Wang HD, Gopalakrishnan V, Wilson TW, McNeill JR Effect of an endothelin antagonist on hemodynamic responses to angiotensin II. Hypertension 28: 806–809, 1996. 8901827 10.1161/01.hyp.28.5.806

[7] Barton M, Tharaux PL Endothelin and the podocyte. Clin Kidney J 5: 17–27, 2012. 26069741 10.1093/ckj/sfs001 PMC4400467

[8] Benigni A, Corna D, Maffi R, Benedetti G, Zoja C, Remuzzi G Renoprotective effect of contemporary blocking of angiotensin II and endothelin-1 in rats with membranous nephropathy. Kidney Int 54: 353–359, 1998. 9690201 10.1046/j.1523-1755.1998.00011.x

[9] Benter IF, Yousif MH, Dhaunsi GS, Kaur J, Chappell MC, Diz DI Angiotensin-(1–7) prevents activation of NADPH oxidase and renal vascular dysfunction in diabetic hypertensive rats. Am J Nephrol 28: 25–33, 2008. 17890855 10.1159/000108758

[10] Boffa JJ, Tharaux PL, Dussaule JC, Chatziantoniou C Regression of renal vascular fibrosis by endothelin receptor antagonism. Hypertension 37: 490–496, 2001. 11230324 10.1161/01.hyp.37.2.490

[11] Bomback AS, Klemmer PJ The incidence and implications of aldosterone breakthrough. Nat Clin Pract Nephrol 3: 486–492, 2007. 17717561 10.1038/ncpneph0575

[12] Buelli S, Rosano L, Gagliardini E, Corna D, Longaretti L, Pezzotta A, Perico L, Conti S, Rizzo P, Novelli R, Morigi M, Zoja C, Remuzzi G, Bagnato A, Benigni A beta-arrestin-1 drives endothelin-1-mediated podocyte activation and sustains renal injury. J Am Soc Nephrol 25: 523–533, 2014. 24371298 10.1681/ASN.2013040362 PMC3935587

[13] Cao Z, Cooper ME, Wu LL, Cox AJ, Jandeleit-Dahm K, Kelly DJ, Gilbert RE Blockade of the renin-angiotensin and endothelin systems on progressive renal injury. Hypertension 36: 561–568, 2000. 11040236 10.1161/01.hyp.36.4.561

[14] Certikova Chabova V, Vernerova Z, Kujal P, Huskova Z, Skaroupkova P, Tesar V, Kramer HJ, Kompanowska-Jezierska E, Walkowska A, Sadowski J, Cervenka L, Vaneckova I Addition of ET(A) receptor blockade increases renoprotection provided by renin-angiotensin system blockade in 5/6 nephrectomized Ren-2 transgenic rats. Life Sci 118: 297–305, 2014. 24373834 10.1016/j.lfs.2013.12.018

[15] Chade AR, Krier JD, Textor SC, Lerman A, Lerman LO Endothelin-a receptor blockade improves renal microvascular architecture and function in experimental hypercholesterolemia. J Am Soc Nephrol 17: 3394–3403, 2006. 17082239 10.1681/ASN.2006060635

[16] Chen L, McNeill JR, Wilson TW, Gopalakrishnan V Heterogeneity in vascular smooth muscle responsiveness to angiotensin II. Role of endothelin. Hypertension 26: 83–88, 1995. 7607737 10.1161/01.hyp.26.1.83

[16a] Clincal Trials.gov. Randomized, double-blind, safety and efficacy study of RE-021 (sparsentan) in focal segmental glomerulosclerosis (DUET). https://clinicaltrials.gov/ct2/show/NCT01613118?term=duet+RE-.

[16b] Clincal Trials.gov. Study of diabetic nephropathy with atrasentan (SONAR). https://clinicaltrials.gov/ct2/show/NCT01858532?term=sonar&rank=.

[17] Daehn I, Casalena G, Zhang T, Shi S, Fenninger F, Barasch N, Yu L, D'Agati V, Schlondorff D, Kriz W, Haraldsson B, Bottinger EP Endothelial mitochondrial oxidative stress determines podocyte depletion in segmental glomerulosclerosis. J Clin Invest 124: 1608–1621, 2014. 24590287 10.1172/JCI71195 PMC3973074

[18] de Zeeuw D, Coll B, Andress D, Brennan JJ, Tang H, Houser M, Correa-Rotter R, Kohan D, Lambers Heerspink HJ, Makino H, Perkovic V, Pritchett Y, Remuzzi G, Tobe SW, Toto R, Viberti G, Parving HH The endothelin antagonist atrasentan lowers residual albuminuria in patients with type 2 diabetic nephropathy. J Am Soc Nephrol 25: 1083–1093, 2014. 24722445 10.1681/ASN.2013080830 PMC4005314

[19] Dhaun N, MacIntyre IM, Bellamy CO, Kluth DC Endothelin receptor antagonism and renin inhibition as treatment options for scleroderma kidney. Am J Kidney Dis 54: 726–731, 2009. 19376621 10.1053/j.ajkd.2009.02.015

[20] Dhaun N, MacIntyre IM, Kerr D, Melville V, Johnston NR, Haughie S, Goddard J, Webb DJ Selective endothelin-A receptor antagonism reduces proteinuria, blood pressure, and arterial stiffness in chronic proteinuric kidney disease. Hypertension 57: 772–779, 2011. 21357275 10.1161/HYPERTENSIONAHA.110.167486

[21] Dhaun N, Macintyre IM, Melville V, Lilitkarntakul P, Johnston NR, Goddard J, Webb DJ Blood pressure-independent reduction in proteinuria and arterial stiffness after acute endothelin-a receptor antagonism in chronic kidney disease. Hypertension 54: 113–119, 2009. 19506099 10.1161/HYPERTENSIONAHA.109.132670

[22] Dhaun N, Macintyre IM, Melville V, Lilitkarntakul P, Johnston NR, Goddard J, Webb DJ Effects of endothelin receptor antagonism relate to the degree of renin-angiotensin system blockade in chronic proteinuric kidney disease. Hypertension 54: e19–e20, 2009. 19652085 10.1161/HYPERTENSIONAHA.109.138263

[23] Emori T, Hirata Y, Ohta K, Kanno K, Eguchi S, Imai T, Shichiri M, Marumo F Cellular mechanism of endothelin-1 release by angiotensin and vasopressin. Hypertension 18: 165–170, 1991. 1909304 10.1161/01.hyp.18.2.165

[24] Emori T, Hirata Y, Ohta K, Shichiri M, Marumo F Secretory mechanism of immunoreactive endothelin in cultured bovine endothelial cells. Biochem Biophys Res Commun 160: 93–100, 1989. 2653322 10.1016/0006-291x(89)91625-2

[25] Gagliardini E, Corna D, Zoja C, Sangalli F, Carrara F, Rossi M, Conti S, Rottoli D, Longaretti L, Remuzzi A, Remuzzi G, Benigni A Unlike each drug alone, lisinopril if combined with avosentan promotes regression of renal lesions in experimental diabetes. Am J Physiol Renal Physiol 297: F1448–F1456, 2009. 19675181 10.1152/ajprenal.00340.2009

[26] Ge Y, Ahn D, Stricklett PK, Hughes AK, Yanagisawa M, Verbalis JG, Kohan DE Collecting duct-specific knockout of endothelin-1 alters vasopressin regulation of urine osmolality. Am J Physiol Renal Physiol 288: F912–F920, 2005. 15632412 10.1152/ajprenal.00432.2004

[27] Gerstung M, Roth T, Dienes HP, Licht C, Fries JW Endothelin-1 induces NF-kappaB via two independent pathways in human renal tubular epithelial cells. Am J Nephrol 27: 294–300, 2007. 17460393 10.1159/000101999

[28] Gilmore ES, Stutts MJ, Milgram SL SRC family kinases mediate epithelial Na+ channel inhibition by endothelin. J Biol Chem 276: 42610–42617, 2001. 11560932 10.1074/jbc.M106919200

[29] Greene E, Kren S, Hostetter T Role of aldosterone in the remnant kidney model in the rat. J Clin Invest 98: 1063–1068, 1996. 8770880 10.1172/JCI118867 PMC507523

[30] Han KH, Kang YS, Han SY, Jee YH, Lee MH, Han JY, Kim HK, Kim YS, Cha DR Spironolactone ameliorates renal injury and connective tissue growth factor expression in type II diabetic rats. Kidney Int 70: 111–120, 2006. 16723984 10.1038/sj.ki.5000438

[31] Herrera M, Hong NJ, Ortiz PA, Garvin JL Endothelin-1 inhibits thick ascending limb transport via Akt-stimulated nitric oxide production. J Biol Chem 284: 1454–1460, 2009. 19033447 10.1074/jbc.M804322200 PMC2615526

[32] Hocher B, Thone-Reineke C, Rohmeiss P, Schmager F, Slowinski T, Burst V, Siegmund F, Quertermous T, Bauer C, Neumayer HH, Schleuning WD, Theuring F Endothelin-1 transgenic mice develop glomerulosclerosis, interstitial fibrosis, and renal cysts but not hypertension. J Clin Invest 99: 1380–1389, 1997. 9077548 10.1172/JCI119297 PMC507954

[33] Hudson M Scleroderma renal crisis. Curr Opin Rheumatol 27: 549–554, 2015. 26352732 10.1097/BOR.0000000000000221

[34] Junaid A, Hostetter TH, Rosenberg ME Interaction of angiotensin II and TGF-β1 in the rat remnant kidney. J Am Soc Nephrol 8: 1732–1738, 1997. 9355076 10.1681/ASN.V8111732

[35] Kashiwagi M, Shinozaki M, Hirakata H, Tamaki K, Hirano T, Tokumoto M, Goto H, Okuda S, Fujishima M Locally activated renin-angiotensin system associated with TGF-β1 as a major factor for renal injury induced by chronic inhibition of nitric oxide synthase in rats. J Am Soc Nephrol 11: 616–624, 2000. 10752520 10.1681/ASN.V114616

[36] Kawaguchi H, Sawa H, Yasuda H Effect of endothelin on angiotensin converting enzyme activity in cultured pulmonary rtery endothelial cells. J Hypertens 9: 171–174, 1991. 1849534 10.1097/00004872-199102000-00012

[37] Kawaguchi H, Sawa H, Yasuda H Endothelin stimulates angiotensin I to angiotensin II conversion in cultured pulmonary artery endothelial cells. J Mol Cell Cardiol 22: 839–842, 1990. 2172556 10.1016/0022-2828(90)90115-i

[38] Kohan DE Endothelin and collecting duct sodium and water transport. Contrib Nephrol 172: 94–106, 2011. 21893992 10.1159/000328687

[39] Kohan DE Endothelin synthesis by rabbit renal tubule cells. Am J Physiol Renal Fluid Electrolyte Physiol 261: F221–F226, 1991. 10.1152/ajprenal.1991.261.2.F221 1877647

[40] Kohan DE, Barton M Endothelin and endothelin antagonists in chronic kidney disease. Kidney Int 86: 896–904, 2014. 24805108 10.1038/ki.2014.143 PMC4216619

[41] Kohan DE, Pritchett Y, Molitch M, Wen S, Garimella T, Audhya P, Andress DL Addition of atrasentan to renin-angiotensin system blockade reduces albuminuria in diabetic nephropathy. J Am Soc Nephrol 22: 763–772, 2011. 21372210 10.1681/ASN.2010080869 PMC3065231

[42] Kohan DE, Rossi NF, Inscho EW, Pollock DM Regulation of blood pressure and salt homeostasis by endothelin. Physiol Rev 91: 1–77, 2011. 21248162 10.1152/physrev.00060.2009 PMC3236687

[43] Kohno M, Horio T, Ikeda M, Yokokawa K, Fukui T, Yasunari K, Kurihara N, Takeda T Angiotensin II stimulates endothelin-1 secretion in cultured rat mesangial cells. Kidney Int 42: 860–866, 1992. 1333547 10.1038/ki.1992.361

[44] Kowala MC, Murugesan N, Tellew J, Carlson K, Monshizadegan H, Ryan C, Gu Z, Kane B, Fadnis L, Baska RA, Beyer S, Arthur S, Dickinson K, Zhang D, Perrone M, Ferrer P, Giancarli M, Baumann J, Bird E, Panchal B, Yang Y, Trippodo N, Barrish J, Macor JE Novel dual action AT1 and ETA receptor antagonists reduce blood pressure in experimental hypertension. J Pharmacol Exp Ther 309: 275–284, 2004. 14718594 10.1124/jpet.103.055855

[45] Laghmani K, Preisig PA, Moe OW, Yanagisawa M, Alpern RJ Endothelin-1/endothelin-B receptor-mediated increases in NHE3 activity in chronic metabolic acidosis. J Clin Invest 107: 1563–1569, 2001. 11413164 10.1172/JCI11234 PMC200190

[46] Lanese DM, Yuan BH, McMurtry IF, Conger JD Comparative sensitivities of isolated rat renal arterioles to endothelin. Am J Physiol Renal Fluid Electrolyte Physiol 263: F894–F899, 1992. 10.1152/ajprenal.1992.263.5.F894 1443178

[47] Lenoir O, Milon M, Virsolvy A, Henique C, Schmitt A, Masse JM, Kotelevtsev Y, Yanagisawa M, Webb DJ, Richard S, Tharaux PL Direct action of endothelin-1 on podocytes promotes diabetic glomerulosclerosis. J Am Soc Nephrol 25: 1050–1062, 2014. 24722437 10.1681/ASN.2013020195 PMC4005294

[48] Licht C, Laghmani K, Yanagisawa M, Preisig PA, Alpern RJ An autocrine role for endothelin-1 in the regulation of proximal tubule NHE3. Kidney Int 65: 1320–1326, 2004. 15086471 10.1111/j.1523-1755.2004.00506.x

[49] Lin H, Sangmal M, Smith MJ Jr, Young DB Effect of endothelin-1 on glomerular hydraulic pressure and renin release in dogs. Hypertension 21: 845–851, 1993. 8500865 10.1161/01.hyp.21.6.845

[50] Mann JF, Green D, Jamerson K, Ruilope LM, Kuranoff SJ, Littke T, Viberti G, Group AS Avosentan for overt diabetic nephropathy. J Am Soc Nephrol 21: 527–535, 2010. 20167702 10.1681/ASN.2009060593 PMC2831858

[51] Matsumura Y, Nakase K, Ikegawa R, Hayashi K, Ohyama T, Morimoto S The endothelium-derived vasoconstrictor peptide endothelin inhibits renin release in vitro. Life Sci 44: 149–157, 1989. 2464732 10.1016/0024-3205(89)90533-x

[52] Mazzocchi G, Malendowicz LK, Meneghelli V, Nussdorfer GG Endothelin-1 stimulates mitotic activity in the zona glomerulosa of the rat adrenal cortex. Cytobios 69: 91–96, 1992. 1317277

[53] Mazzocchi G, Rebuffat P, Meneghelli V, Malendowicz LK, Kasprzak A, Nussdorfer GG Effects of prolonged infusion with endothelin-1 on the function and morphology of rat adrenal cortex. Peptides 11: 767–772, 1990. 2172942 10.1016/0196-9781(90)90193-9

[54] Murugesan N, Tellew JE, Gu Z, Kunst BL, Fadnis L, Cornelius LA, Baska RA, Yang Y, Beyer SM, Monshizadegan H, Dickinson KE, Panchal B, Valentine MT, Chong S, Morrison RA, Carlson KE, Powell JR, Moreland S, Barrish JC, Kowala MC, Macor JE Discovery of N-isoxazolyl biphenylsulfonamides as potent dual angiotensin II and endothelin A receptor antagonists. J Med Chem 45: 3829–3835, 2002. 12190306 10.1021/jm020138n

[55] Nadler SP, Zimpelmann JA, Hebert RL Endothelin inhibits vasopressin-stimulated water permeability in rat terminal inner medullary collecting duct. J Clin Invest 90: 1458–1466, 1992. 1328300 10.1172/JCI116013 PMC443192

[56] Opocensky M, Dvorak P, Maly J, Kramer HJ, Backer A, Kopkan L, Vernerova Z, Tesar V, Zima T, Bader M, Ganten D, Janda J, Vaneckova I Chronic endothelin receptor blockade reduces end-organ damage independently of blood pressure effects in salt-loaded heterozygous Ren-2 transgenic rats. Physiol Res 53: 581–593, 2004. 15588125

[57] Otsuka A, Mikami H, Katahira K, Tsunetoshi T, Minamitani K, Ogihara T Changes in plasma renin activity and aldosterone concentration in response to endothelin injection in dogs. Acta Endocrinol 121: 361–364, 1989. 2678869 10.1530/acta.0.1210361

[58] Parvanova A, van der Meer IM, Iliev I, Perna A, Gaspari F, Trevisan R, Bossi A, Remuzzi G, Benigni A, Ruggenenti P, Daglutril in Diabetic Nephropathy Study Group. Effect on blood pressure of combined inhibition of endothelin-converting enzyme and neutral endopeptidase with daglutril in patients with type 2 diabetes who have albuminuria: a randomised, crossover, double-blind, placebo-controlled trial. Lancet Diabetes Endocrinol 1: 19–27, 2013. 24622263 10.1016/S2213-8587(13)70029-9

[59] Pavlov TS, Chahdi A, Ilatovskaya DV, Levchenko V, Vandewalle A, Pochynyuk O, Sorokin A, Staruschenko A Endothelin-1 inhibits the epithelial Na+ channel through betaPix/14-3-3/Nedd4-2. J Am Soc Nephrol 21: 833–843, 2010. 20338996 10.1681/ASN.2009080885 PMC2865733

[60] Rakugi H, Nakamaru M, Saito H, Higaki J, Ogihara T Endothelin inhibits renin release from isolated rat glomeruli. Biochem Biophys Res Commun 155: 1244–1247, 1988. 3052444 10.1016/s0006-291x(88)81273-7

[61] Riggleman A, Harvey J, Baylis C Endothelin mediates some of the renal actions of acutely administered angiotensin II. Hypertension 38: 105–109, 2001. 11463769 10.1161/01.hyp.38.1.105 PMC2745261

[62] Rossi GP, Ganzaroli C, Cesari M, Maresca A, Plebani M, Nussdorfer GG, Pessina AC Endothelin receptor blockade lowers plasma aldosterone levels via different mechanisms in primary aldosteronism and high-to-normal renin hypertension. Cardiovasc Res 57: 277–283, 2003. 12504838 10.1016/s0008-6363(02)00658-2

[63] Ruster C, Wolf G Angiotensin II as a morphogenic cytokine stimulating renal fibrogenesis. J Am Soc Nephrol 22: 1189–1199, 2011. 21719784 10.1681/ASN.2010040384

[64] Saleh MA, Boesen EI, Pollock JS, Savin VJ, Pollock DM Endothelin receptor A-specific stimulation of glomerular inflammation and injury in a streptozotocin-induced rat model of diabetes. Diabetologia 54: 979–988, 2011. 21191784 10.1007/s00125-010-2021-4 PMC3804244

[65] Saleh MA, Boesen EI, Pollock JS, Savin VJ, Pollock DM Endothelin-1 increases glomerular permeability and inflammation independent of blood pressure in the rat. Hypertension 56: 942–949, 2010. 20823379 10.1161/HYPERTENSIONAHA.110.156570 PMC2959121

[66] Saleh MA, Pollock JS, Pollock DM Distinct actions of endothelin A-selective versus combined endothelin A/B receptor antagonists in early diabetic kidney disease. J Pharmacol Exp Ther 338: 263–270, 2011. 21471190 10.1124/jpet.111.178988 PMC3126640

[67] Saleh MA, Sandoval RM, Rhodes GJ, Campos-Bilderback SB, Molitoris BA, Pollock DM Chronic endothelin-1 infusion elevates glomerular sieving coefficient and proximal tubular albumin reuptake in the rat. Life Sci 91: 634–637, 2012. 22727794 10.1016/j.lfs.2012.06.007 PMC3728660

[68] Sasser JM, Murphy SR, Granger JP Emerging drugs for preeclampsia–the endothelium as a target. Expert Opin Emerg drugs 20: 527–530, 2015. 26138471 10.1517/14728214.2015.1062875 PMC4697827

[69] Sasser JM, Sullivan JC, Hobbs JL, Yamamoto T, Pollock DM, Carmines PK, Pollock JS Endothelin A receptor blockade reduces diabetic renal injury via an anti-inflammatory mechanism. J Am Soc Nephrol 18: 143–154, 2007. 17167119 10.1681/ASN.2006030208 PMC2579758

[70] Schneider MP, Ge Y, Pollock DM, Pollock JS, Kohan DE Collecting duct-derived endothelin regulates arterial pressure and Na excretion via nitric oxide. Hypertension 51: 1605–1610, 2008. 18391099 10.1161/HYPERTENSIONAHA.107.108126 PMC3799791

[71] Shanmugam VK, Steen VD Renal disease in scleroderma: an update on evaluation, risk stratification, pathogenesis and management. Curr Opin Rheumatol 24: 669–676, 2012. 22955019 10.1097/BOR.0b013e3283588dcf PMC4048657

[72] Simonson MS, Wann S, Mené P, Dubyak GR, Kester M, Nakazato Y, Sedor JR Endothelin stimulates phospholipase C, Na+/H+ exchange, c-fos expression, and mitogenesis in rat mesangial cells. J Clin Invest 83: 708–712, 1989. 2536405 10.1172/JCI113935 PMC303732

[73] Simonson MS, Ismail-Beigi F Endothelin-1 increases collagen accumulation in renal mesangial cells by stimulating a chemokine and cytokine autocrine signaling loop. J Biol Chem 286: 11003–11008, 2011. 21169360 10.1074/jbc.M110.190793 PMC3064155

[74] Sun GP, Kohno M, Guo P, Nagai Y, Miyata K, Fan YY, Kimura S, Kiyomoto H, Ohmori K, Li DT, Abe Y, Nishiyama A Involvements of Rho-kinase and TGF-beta pathways in aldosterone-induced renal injury. J Am Soc Nephrol 17: 2193–2201, 2006. 16790507 10.1681/ASN.2005121375

[75] Tharaux PL Endothelin in renal injury due to sickle cell disease. Contrib Nephrol 172: 185–199, 2011. 21893999 10.1159/000328699

[76] Vallon V, Wyatt AW, Klingel K, Huang DY, Hussain A, Berchtold S, Friedrich B, Grahammer F, Belaiba RS, Gorlach A, Wulff P, Daut J, Dalton ND, Ross J Jr, Flogel U, Schrader J, Osswald H, Kandolf R, Kuhl D, Lang F SGK1-dependent cardiac CTGF formation and fibrosis following DOCA treatment. J Mol Med 84: 396–404, 2006. 16604333 10.1007/s00109-005-0027-z

[77] Wallukat G, Homuth V, Fischer T, Lindschau C, Horstkamp B, Jupner A, Baur E, Nissen E, Vetter K, Neichel D, Dudenhausen JW, Haller H, Luft FC Patients with preeclampsia develop agonistic autoantibodies against the angiotensin AT1 receptor. J Clin Invest 103: 945–952, 1999. 10194466 10.1172/JCI4106 PMC408252

[78] Watson AM, Li J, Schumacher C, de Gasparo M, Feng B, Thomas MC, Allen TJ, Cooper ME, Jandeleit-Dahm KA The endothelin receptor antagonist avosentan ameliorates nephropathy and atherosclerosis in diabetic apolipoprotein E knockout mice. Diabetologia 53: 192–203, 2010. 19862499 10.1007/s00125-009-1540-3

[79] Webb ML, Dickinson KE, Delaney CL, Liu EC, Serafino R, Cohen RB, Monshizadegan H, Moreland S The endothelin receptor antagonist, BQ-123, inhibits angiotensin II-induced contractions in rabbit aorta. Biochem Biophys Res Commun 185: 887–892, 1992. 1320879 10.1016/0006-291x(92)91710-8

[80] Weber MA, Black H, Bakris G, Krum H, Linas S, Weiss R, Linseman JV, Wiens BL, Warren MS, Lindholm LH A selective endothelin-receptor antagonist to reduce blood pressure in patients with treatment-resistant hypertension: a randomised, double-blind, placebo-controlled trial. Lancet 374: 1423–1431, 2009. 19748665 10.1016/S0140-6736(09)61500-2

[81] Wennmann DO, Hsu HH, Pavenstadt H The renin-angiotensin-aldosterone system in podocytes. Semin Nephrol 32: 377–384, 2012. 22958492 10.1016/j.semnephrol.2012.06.009

[82] Wenzel RR, Littke T, Kuranoff S, Jurgens C, Bruck H, Ritz E, Philipp T, Mitchell A, SPP301 (Avosentan) Endothelin Antagonist Evaluation in Diabetic Nephropathy Study Investigators. Avosentan reduces albumin excretion in diabetics with macroalbuminuria. J Am Soc Nephrol 20: 655–664, 2009. 19144760 10.1681/ASN.2008050482 PMC2653691

[83] Wenzel RR, Ruthemann J, Bruck H, Schafers RF, Michel MC, Philipp T Endothelin-A receptor antagonist inhibits angiotensin II and noradrenaline in man. Br J Clin Pharmacol 52: 151–157, 2001. 11488771 10.1046/j.0306-5251.2001.01422.x PMC2014518

[84] Wong S, Brennan FE, Young MJ, Fuller PJ, Cole TJ A direct effect of aldosterone on endothelin-1 gene expression in vivo. Endocrinology 148: 1511–1517, 2007. 17218419 10.1210/en.2006-0965

[85] Yang F, Chung AC, Huang XR, Lan HY Angiotensin II induces connective tissue growth factor and collagen I expression via transforming growth factor-beta-dependent and -independent Smad pathways: the role of Smad3. Hypertension 54: 877–884, 2009. 19667256 10.1161/HYPERTENSIONAHA.109.136531

[86] Zoja C, Benigni A, Remuzzi G Protein overload activates proximal tubular cells to release vasoactive and inflammatory mediators. Exp Nephrol 7: 420–428, 1999. 10559640 10.1159/000020640

[87] Zoja C, Cattaneo S, Fiordaliso F, Lionetti V, Zambelli V, Salio M, Corna D, Pagani C, Rottoli D, Bisighini C, Remuzzi G, Benigni A Distinct cardiac and renal effects of ETA receptor antagonist and ACE inhibitor in experimental type 2 diabetes. Am J Physiol Renal Physiol 301: F1114–F1123, 2011. 21816757 10.1152/ajprenal.00122.2011

